# Metabolic Disorders in Multiple Myeloma

**DOI:** 10.3390/ijms222111430

**Published:** 2021-10-22

**Authors:** Maria Gavriatopoulou, Stavroula A. Paschou, Ioannis Ntanasis-Stathopoulos, Meletios A. Dimopoulos

**Affiliations:** Department of Clinical Therapeutics, School of Medicine, National and Kapodistrian University of Athens, 11528 Athens, Greece; spaschou@med.uoa.gr (S.A.P.); johnntanasis@med.uoa.gr (I.N.-S.); mdimop@med.uoa.gr (M.A.D.)

**Keywords:** multiple myeloma, metabolism, glycolysis, glutaminolysis, metabolic syndrome

## Abstract

Multiple myeloma (MM) is the second most common hematological malignancy and is attributed to monoclonal proliferation of plasma cells in the bone marrow. Cancer cells including myeloma cells deregulate metabolic pathways to ensure proliferation, growth, survival and avoid immune surveillance, with glycolysis and glutaminolysis being the most identified procedures involved. These disorders are considered a hallmark of cancer and the alterations performed ensure that enough energy is available for rapid cell proliferation. An association between metabolic syndrome, inflammatory cytokinesand incidence of MM has been also described, while the use of metformin and statins has been identified as a positive prognostic factor for the disease course. In this review, we aim to present the metabolic disorders that occur in multiple myeloma, the potential defects on the immune system and the potential advantage of targeting the dysregulated pathways in order to enhance antitumor therapeutics.

## 1. Introduction

Multiple myeloma (MM) is the second most common hematologic malignancy and is attributed to bone marrow infiltration by monoclonal plasma cells. The plasma cell proliferation in the marrow leads to increased production and circulation of the monoclonal paraprotein (M-spike) in serum and/or urine [1]. The cardinal clinical features of MM include anemia, hypercalcemia, renal impairmentand myeloma-related bone lesions [2]. The prognosis of MM patients has significantly improved over the past 15 years, mainly due to the incorporation of novel agents.However, it still remains an incurable disease and all patients will eventually relapse [3]. MM is considered a disease of the elderly, with a median onset age of 69 years. The elderly population presents usually with concurrent co-morbidities such as obesity, diabetes and hyperlipidemia [4,5,6,7,8,9]. Possibly, these metabolic disorders might be involved in the disease pathogenesis, although it has not yet been clearly defined. Although the exact underlying pathogenetic mechanisms of the disease are unknown, it has been demonstrated that the disease is more common in African Americans. On the other hand, this subgroup of patients has a better prognosis when compared with matched Caucasian patients [10,11]. These racial disparities have been correlated with racial variations in gene loci associated with inflammation [12,13]. Furthermore, other functional gene loci with ethnic variations among myeloma patients are implicated in several metabolic pathways in MM [14].

Metabolic disorders are considered a hallmark of cancer and the changes that occur in metabolic pathways are necessary to ensure that enough energy is available for rapid cell proliferation and tumor growth. In this context, glycolysis and glutaminolysis are the two main metabolic pathways that are deregulated and might be combined with immune system impairment [15,16]. It has to be noted that the patterns of metabolic landscape in myeloma cells are dynamic during the disease course. Metabolic changes have been associated with acquired resistance to backbone treatment agents in myeloma such as bortezomib and melphalan [17,18,19]. Interestingly, a heterogenous metabolic pattern in baseline positron emission tomography/computed tomography (PET/CT) has been recently associated with adverse prognosis in patients with MM [20]. Therefore, response to antimyeloma treatment might be indirectly correlated to impaired metabolic status and changes in microenvironment [21,22]. Furthermore, metabolic signatures may have a prognostic value in patients with MM. A recent study has formulated a validated prognostic model based on the expression status of seven genes related to metabolic pathways. Patients belonging in the high-risk metabolic group had a significantly worse survival rate compared with those presenting with a low-risk metabolic profile (62% versus 85%, respectively, *p* < 0.001) [23].

**Figure 1:**
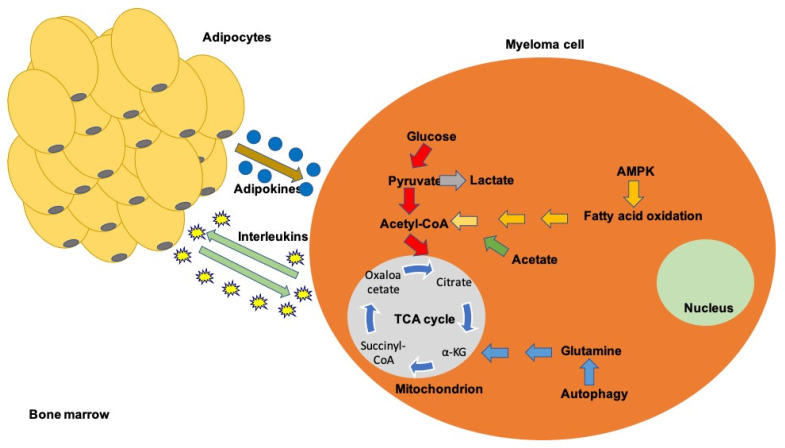
A schematic overview of the most important metabolic disturbances in myeloma microenvironment.

In this review, we aim to present the metabolic disorders that occur in multiple myeloma, the potential defects on the immune system and the potential advantage of targeting the dysregulated pathways in order to enhance antitumor therapeutics (Figure 1).

## 2. Glucose and Glutamine Pathways-General Principles

The main metabolic components involved in multiple myeloma pathogenesis are the glucose and glutamine pathways. Hexokinase II is an enzyme overexpressed in multiple myeloma that plays the role of a catalyst in the initial steps of glucose metabolism [24,25]. In combination with other metabolites and enzymes involved in the glucose pathway, they could be potential targets to reverse the increased glycolysis in myeloma cells. The glutamine pathway can also be targeted to alter the increased metabolic activities [26]. Additionally, the role of the folate and proline pathways in multiple myeloma metabolism are currently under investigation. Myeloma cells undergo several metabolic changes such as rearrangements involving adjustments in glucose, glutamine pentose phosphate, folate pathway and serine metabolism. These alterations might be involved in drug resistance along with hypoxia, apoptosis inhibition, epigenetic effects, drug inactivation, DNA damage repair and drug efflux [27]. For example, resistance to bortezomib, a well-known and widely used proteasome inhibitor has been correlated with the upregulation of several metabolic pathways that could be potentially targeted. Resistance to immunotherapeutic agents resistance has also been linked to metabolic disorders. Novel compounds targeting metabolic pathways, if combined with immunotherapy along with the available treatment options, might overcome the induced resistance [28].

## 3. Glycolysis and Gluconeogenesis

In contrast to normal cells, cancer cells and myeloma cells depend on aerobic glycolysis which converts glucose into lactic acid. This conversion leads to adenosine triphosphate (ATP) production that is necessary for their growth and survival. It is known that myeloma cells depend on glycolysis and are vulnerable to glycolysis inhibitors such as inhibitors of glucose transporter (GLUT) and key glycolytic enzymes [29]. The upregulation of GLUT1 isoform elevates glucose uptake and therefore GLUT1-inhibition can potentially induce myeloma cell death [30]. Furthermore, other isoforms such as GLUT4 are also crucial for myeloma growth and proliferation [31]. Initially, glucose is transported in the cells, transformed into lactate which induces ATP production. This is mediated by hexokinase 2 (HK2), phosphofructokinase (PFK), pyruvate kinase M2 (PKM2) and lactate dehydrogenase A (LDHA) that are highly expressed in myeloma [28]. An upregulation of genes associated with aerobic glycolysis such as PKM2 and NIMA related kinase 2 (NEK2) has been associated with inferior survival outcomes in patients with MM [32]. Along with glycolysis the pentose phosphate bypass pathway (PPP) is also activated leading to increased production of nicotinamide adenine dinucleotide phosphate (NADPH) and glutathione (GSH) that is known to support tumor cells against oxidative stress [33]. It is well known that oxidative stress is one of the main mechanisms targeted by bortezomib and this might explain the drug resistance that is linked to increased antioxidant capacity. Currently available data show that the production of NADPH may induce proteasome inhibitors intolerance [34]. Lactate is transported by the monocarboxylate transporters (MCT) MCT1 and MCT4. The expression of MCT1 is increased under aerobic conditions, while MCT4 is increased under hypoxia [35].

Gluconeogenesis is a two-step process that leads to glucose production, therefore ensuring stable blood levels. Pyruvate is initially converted to oxaloacetate and finally converted to phosphoenolpyruvic acid (PEP). Subsequently, PEP is converted and phosphorylated to 1,2-bisphosphoglycerate. Several metabolic steps lead to the final product, which is glucose 6-phosphatase that dephosphorylates forming glucose. This procedure is considered the opposite of glycolysis and both share same enzymes and regulators to ensure the homeostasis of blood glucose levels [28].

## 4. Glutaminolysis

Glutamine is another important component in plasma cell metabolism since depletion of glutamine limits myeloma cell growth [36]. Glutamine is important for amino acids and nucleotides synthesis [37]. Bortezomib-resistant myeloma cell lines have shown increased mitochondrial function enhanced mainly by glutamine compared to glucose. Therefore, alterations in glutamine metabolism might be involved both in treatment efficacy and drug resistance [38]. Glutaminase inhibitors may restore the efficacy of proteasome inhibitors by restoring caspase-mediated death signals [39]. c-Myc increases glutamine transporters and glutaminase (GLS) expression leading to enhanced glutaminolysis [40]. In glutaminolysis, glutamine is transported in the cells by binding to glutamine transporters such as amino acid transporter ASCT2 (SLC1A5) and SNAT1 (SLC38A1) [41], is converted to glutamate and α-ketoglutarate (α-KG) enhancing the TCA cycle [42,43]. It has been demonstrated that CD138+ cells upregulate the expression of the glutamine transporters ASCT2 (SLC1A5), LAT1 (SLC7A5) and SNAT1. However, the ASCT2 inhibition is the only one identified that decreases glutamine uptake and myeloma cell growth [44].

## 5. The TCA Cycle

The TCA cycle is the main catabolic pathway that mediates the production of ATP, which is the main energy component for myeloma cells. TCA cycle enhances this procedure through fatty and amino acids metabolism along with the presence of oxygen. Based on the energy needs of each cell the TCA cycle has several different regulation mediators that might enhance or down regulate the relevant procedures [45]. Patients with MM present higher levels of TCA intermediates compared with patients with the premalignant state of monoclonal gammopathy of undetermined significance (MGUS) [46]. Increased glutamine anaplerosis into the TCA cycle in myeloma cells is associated with increased expression of the proto-oncogene c-myc. C-myc mutations are considered among the driver events of progression from MGUS to MM [47].

## 6. Fatty and Amino Acid Synthesis

Fatty acids are responsible for stabilizing cell membranes and are important components of long-term energy reserves. The synthesis starts with the reaction between acetyl-CoA and malonyl-ACP, leading to acetoacetyl-ACP and C02, finally ending to synthesis of glycerophospholipids, triacylglycerides, phosphatidateand phosphatidylcholine. The production as well as the degradation of fatty acids is mainly dependent on the energy reservoir. Whenever increased energy is required, ATP molecules are produced to accelerate fatty acids degradation [28]. Amino acids are involved in protein and hormone synthesis. In cases of energy starvation, fatty acids are exhausted and then proteins are degraded to balance the energy gap. Protein oxidation leads to L-glutamine that is transferred to liver to produce energy. Alternatively, the glucose-alanine cycle is activated to metabolize the protein products [28]. Both increased fatty acid and amino acid synthesis may result from increased glutaminolysis and promote cancer cell survival [48]. Furthermore, fatty acids derived from bone marrow adipocytes may sustain the survival and propagation of myeloma cells [49]. Myeloma cells have also shown to increase fatty acid binding proteins (FABPs), which may promote tumor growth [50,51]. Interestingly, pharmacological inhibition of fatty acid metabolism by etomoxir, which inhibits fatty acid beta oxidation, and orlistat, which inhibits de novo fatty acid synthesis, suspended myeloma proliferation and reduced myeloma cell survival [52].

## 7. Immune Cells Metabolic Disorders

As already described, cancer cells undergo metabolic alterations; however, it seems that such alterations occur in immune cells as well, and this may lead to tumor survival through immune escape [53,54]. M1 macrophages induce anabolic procedures, such as glycolysis and fatty acid biosynthesis (FAS) and M2 macrophages induce oxidative phosporylation (OXPHOS). Aerobic glycolysis is mainly induced by toll like receptor (TLR)-induced signaling that stabilizes hypoxia-inducible factor (HIF)-1α and boosts mammalian target of rapamycin (mTOR) [55]. Similarly, naïve and memory T cells mainly depend on OXPHOS and fatty acid β-oxidation (FAO) leading to ATP production [56,57]. Post-activation, T-effector cells switch to glycolysis and glutaminolysis suppressing FAO [58]. This is mediated by T cell receptor (TCR) and CD28 by phosphatidylinositol 3′-kinase (PI3K)-AKT-mTOR pathway activation and upregulation of metabolic enzymes [59]. Activated NK cells also induce glycolysis, since PI3K is required for NK maturation, homing and functioning [60,61]. Since immune cells share the same metabolic requirements with myeloma cells, metabolic competition is created. During the disease course from asymptomatic to symptomatic and relapsing myeloma, the immune microenvironment is highly dynamic and changes in the immune signatures also result in changes in the metabolic profile [62]. Cancer cells, through hypoxia and lactate increase, have better access to nutrients, which promotes tumor survival and inhibits immune surveillance [63]. Based on the available data, it is possible that controlling the myeloma cells could also restore immune cells’ functionality. Novel antimyeloma treatments and autologous stem cell transplantation might reduce CD4+ naïve T cells, increase CD8+ memory T cells and increase CD4+ T cells that overexpress PD1 [64]. The understanding of immune cell restoration might lead to more effective treatment strategies in the near future.

## 8. PI3K-AKT and AMPK Pathways

The PI3K-Akt signaling pathway regulates transcription, translation, proliferation, growth and survival. In MM, it is activated by interleukin (IL)-6 [65,66] and stromal-derived factor (SDF)-1 [67,68]. Following activation, Akt promotes mTOR and mTORC1 enhances the expression of glycolytic enzymes such as PFK leading to increased glycolysis [69].

Adenosine monophosphate (AMP)-activated protein kinase (AMPK) is an energy sensor of cellular activities and identifies energy stress by upregulating signal transduction pathways and downregulating ATP-consuming biosynthesis processes. Furthermore, AMPK suppresses the mammalian target of rapamycin (mTOR) signaling pathway [70]. Upregulation of the AMPK could be proven beneficial for antimyeloma treatment. Preclinical studies have shown that resveratrol induces the phosphorylation of AMPK, which in turn decreases the phosphorylation of mTOR and its downstream targets, that leads myeloma cells to autophagy [71].

## 9. The Transcription Factors HIF-1α, c-MYC, and P53

HIF-1α is highly expressed in myeloma bone marrow and is considered as a regulator of cellular metabolism [72]. HIF-1α enhances the expression of glycolytic genes and suppressors of the TCA cycle [73,74]. Increased expression of HIF-1α may lead to drug resistance [19,75]. Interestingly, pharmacologic inhibition of HIF-1α is able to restore sensitivity to bortezomib in myeloma cell lines [19]. MYC family is an oncogene family that regulates the genes involved in glycolysis and glutaminolysis [76]. When combined with HIF it leads to mitochondrial impairment. P53 is a tumor suppressor and the deletion or mutation of the TP53 gene is considered as one of the most important negative prognostic factors in MM [77]. P53 is among the key regulators of cancer metabolism [78,79,80]. P53 induces aerobic glycolysis and inhibits the PI3K-Akt pathway [80]. Preclinical MM models with P53 mutations have shown that BCMA overexpression alters the metabolic profile and induces an immunosuppressive phenotype in the bone marrow in this setting [81].

## 10. Metabolic Deregulation of Angiogenesis

Increased angiogenesis in patients with multiple myeloma induces tumor proliferation. This is mediated mainly through vascular endothelial growth factor (VEGF) and fibroblast growth factor-2 (FGF-2) and is correlated with worse prognosis. The endothelial marrow cells secrete interleukin-6 and promote additional myeloma cell growth. Increased angiogenesis reverses hypoxia and therefore the potential use of anti-angiogenic agents results in a more hypoxic environment, along with increased glycolysis [82,83,84]. Other interleukins such as IL-6 and IL-3 are also involved promoting myeloma cell differentiation [85]. Furthermore, myeloma cells impair the bone marrow microenvironment ensuring their survival. More specifically, tumor cells adhere to stromal cells, activate several antiapoptotic pathways such as Janus kinase (JAK)/signal transducer and activator of transcription 3 (STAT3) and upregulate anti-apoptotic proteins such as BcL-xL, nuclear factor-κB (NF-κB) and Mcl-1 [86]. The abovementioned interactions may lead to anemia via their adverse effect on the erythropoietic niches [87].

## 11. Metabolic Disturbances of Calcium Metabolism

Hypercalcemia is a common metabolic disorder in multiple myeloma patients, especially those with high tumor burden and aggressive disease forms such as plasma cell leukemia [88]. The main reason is the increased osteoclastic bone resorption compared with renal impairment if present. In this case, the kidney functionality to clear calcium load is downregulated, thus resulting in increased serum calcium levels [89]. Bone metabolism is markedly deregulated in MM [90,91]. The bone resorption is mediated by receptor activator of NF-κB ligand [RANKL], macrophage inflammatory protein [MIP]-1α, and tumor necrosis factors [TNFs]. The relevant cytokines are secreted either by myeloma cells or in the bone marrow microenvironment [92]. Myeloma cells activate the osteoclasts leading to bone resorption. The upregulation of the osteoclasts is mediated by adhesion modules such as vascular cell adhesion molecule 1 (VCAM-1) and α4β1 integrin. Additionally, the RANKL induces osteolysis along with other osteoclast-activating factors [93,94]. The induced bone resorption leads to calcium in the extracellular fluid. Other factors seem to be involved in the pathogenesis of hypercalcemia; therefore, further investigation is required. Parathyroid hormone-related protein (PTHrP) is usually within normal limits in contrary with solid tumors and myeloma patients usually respond rapidly to corticosteroids administration, due to rapid control of the disease. Interestingly, anti-myeloma treatment seems to restore bone metabolism [95,96,97,98,99], whereas anti-resorptive treatment such as bisphosphonates and anti-RANKL agents reverse myeloma-induced hypercalcemia [90,96].

## 12. Metabolic Syndrome

Metabolic syndrome represents a cluster of clinical features including central obesity, insulin resistance and hyperglycemia, dyslipidemia and hypertension [89]. There is increasing evidence for higher prevalence of the syndrome or its individual components in patients with MM [100]. The question arising is whether this phenomenon is due to the advanced age of MM patients, due to the disease itself or due to antimyelomaincluding steroids, chemotherapy, and bone marrow transplantation [100,101]. Obesity has been identified by several studies as risk factor for MM development and increased mortality [102,103,104,105,106]. A meta-analysis, including 19 prospective studies [103] reported a statistically significant increased incidence of MM in overweight individuals (body mass index, BMI > 25 kg/m^2^, risk ratio, RR = 1.12) or obese individuals (BMI > 30 kg/m^2^, RR = 1.21) compared to controls with normal weight. Moreover, RR estimates of MM mortality were 1.15 and 1.54 for overweight and obese patients, respectively [103]. A case study of 82 patients at various stages of MM reported that 58.5% of patients had at least one feature of metabolic syndrome [97]. A more advanced approach to study the possible effect of metabolic syndrome on MM risk accounts for confounding variables such as environmental and dietary factors [107,108,109,110,111,112,113]. Mendelian randomization studies have shown contradictory results. Although the two prior studies have not shown a significant correlation between obesity/adiposity and risk of MM [114,115], a recent study revealed a possible causal relationship between MM and greater genetically instrumented unfavorable adiposity according to single nucleotide polymorphisms [116].

In addition to the above, obesity increases the number and size of bone marrow adipocytes [117]. Accumulating evidence has shown that adipocytes are in close interplay with myeloma cells [117,118]. Bone marrow adipocytes have a dual role; being an energy depository andinvolvement in metabolic activity by providing adipokines and bioactive molecules [118]. Myeloma cells may regulate adipogenesis in order to sustain their survival and homing in the bone marrow milieu [118]. Myeloma cells induce the expression of genes related to an inflammatory state and a senescence associated secretory phenotype (SAPS) in the adipocytes, in order to promote myelomatogenesis and myeloma cell survival [119]. Interestingly, the response to treatment may restore the adipocyte homeostasis in the bone marrow [120].

Several explanations have been proposed for the association of central obesity with MM. Insulin resistance, hyperinsulinemia and subsequent hyperglycemia, overproduction of insulin like growth factor 1 (IGF-1) and increased secretion of inflammatory cytokines are considered as the most important factors related [101]. Interestingly, there have been reported some cases of patients with MM who develop hypoglycemia episodes even in the absence of a prior history of diabetes mellitus (DM). In these cases, hypoglycemia has been associated with the ability of monoclonal paraprotein to recognize and bind to insulin [121,122,123]. These anti-insulin monoclonal antibodies have low affinity to insulin. Therefore, insulin may be able to bind to its receptor, but it has a delayed clearance and a prolonged effect on glucose homeostasis [121].

A retrospective review of 1240 MM patients investigated the rate of DM, as well as the impact of DM and anti-diabetic therapies on MM clinical outcomes [124]. Type 2 DM and steroid-induced DM were present in 12.6% and 31.7% of the cohort, respectively. Patients with DM presented a significantly reduced overall survival (OS) (median 65.4 months) compared to non-diabetic patients (median 98.7 months). In the multivariate analysis, steroid-induced DM was found as a significant predictor of poor OS [124]. Interestingly, there were differences among anti-diabetic medications. Specifically, metformin use was associated with decreased mortality, while insulin analogues were associated with increased mortality [124]. Furthermore, preclinical studies have shown that metformin amplifies DNA damage induced by melphalan. Metformin decreases the availability of ATP for the damage repair response in myeloma cells, which in turn leads them in increased apoptosis [125]. Other studies also reported high prevalence of DM, between 18 and 22%, already at the time of MM diagnosis [126,127]. Moreover, the results of a recent study demonstrated a higher prevalence of DM (25%) among individuals even with smoldering MM (SMM) compared to a healthy control group (8%) at baseline [101].

Dyslipidemia has been also extensively described in patients with MM, and particularly in those with the immunoglobulin (Ig) A subtype [100,128,129]. It can be present as isolated hypercholesterolemia or hypertriglyceridemia or in combination with clinical features, such as cutaneous xanthomas and hyperviscosity syndrome [128,129]. The results of a recent study demonstrated a higher prevalence of dyslipidemia (54%) among individuals even with SMM compared to a healthy control group (32%) [101]. Interestingly, several case reports of acquired myeloma-related Frederickson’s type III hyperlipoproteinemia (increased remnant or intermediate-density lipoproteins, IDL) and type V hyperlipoproteinemia (increased chylomicron and very low-density lipoproteins, VLDL) hyperlipoproteinemia have been published [130,131]. Many studies have implicated paraproteins in the development of MM associated dyslipidemia [101,129,132]. The binding of paraproteins to lipoprotein lipase, serum lipoproteins or tissue receptors, may result in clearance reduction of lipoproteins and could explain the phenomenon from a pathophysiological point of view [100,132]. Additionally, the fractional catabolic rate of IDL, the conversion of IDL to low density lipoproteins (LDL), and the affinity of LDL to its receptor have been found to be impaired in patients with MM, possibly due to the formation of Ig-lipoprotein complexes [100]. LDL may prevent myeloma cell apoptosis and promote myeloma cell survival [133]. Interestingly, patients with MM and high APOA1 serum levels, which is the major component of high density lipoprotein (HDL), present superior survival outcomes compared with patients with low APOA1 levels [134,135]. Patients with MM have lower levels of HDL-C and higher levels of triglycerides at diagnosis and when myeloma is active, as compared with healthy individuals [136]. Furthermore, patients with favorable disease traits (international staging system, ISS-1) may present with higher HDL levels compared with those with adverse prognostic characteristics (ISS-3) [134,135,137]. Dyslipidemia in patients with MM may be relatively refractory to conventional lipid lowering medications, but it seems responsive when MM is successfully treated [100,138]. On the other hand, there is accumulating recent evidence to suggest that statin use may reduce both the risk of MM development and mortality [139,140]. Interestingly, the combination of lipid-lowering agents with proteasome inhibitors has shown to have a synergistic effect against myeloma cells [141].

Hypertension is another component of metabolic syndrome and it has been reported to be present with high incidence in patients with MM [142,143]. Additionally, there are increased events of malignant hypertension in individuals with MM. It is possible that certain therapies used in MM such as high-dose steroids and selective proteasome inhibitors, such as carfilzomib, may evoke hypertension in some patients [144,145]. However, even at baseline and before any treatment initiation, the prevalence of hypertension among MM patients approaches 38–47% [142,146]. The results of a recent study demonstrated a higher prevalence of hypertension (60%) among individuals even with SMM compared to a healthy control group (41%) [101].

## 13. Myeloma Treatment and Metabolism

The treatment landscape in MM includes several anti-myeloma agents and drug combinations with distinct mechanisms of action. The main drug categories include proteasome inhibitors (bortezomib, carfilzomib, ixazomib), immunomodulatory drugs (lenalidomide, pomalidomide, thalidomide), anti-CD38 monoclonal antibodies (daratumumab, isatuximab), anti-SLAMF7 monoclonal antibodies (elotuzumab), epigenetic modifiers (panobinostat), conjugated antibodies (belantamabmafodotin) and selective inhibitors of nuclear export (selinexor) [147]. Preclinical studies have shown that anti-myeloma drugs exert a selective pressure on myeloma cells, which have increased energy demands for survival. Myeloma cells can hijack mitochondria from nearby bone marrow mesenchymal stem cells through partial cell fusion and tunneling nanotubes [148]. Therefore, myeloma cells acquire resistance to chemotherapeutic drugs. Interestingly, anti-mitochondrial agents have shown to restore the sensitivity of myeloma cells to proteasome inhibitors [39]. The combination of metabolic regulators with proteasome inhibitors may induce synthetic lethality, prevent the activation of resistance mechanisms and increase efficacy [141,149]. Recently, preclinical studies have shown that adaptive natural killer cells have decreased CD38 expression and enhanced metabolic fitness by resisting oxidative stress, which may lead to improved anti-myeloma activity in the relapsed disease setting [150,151]. Furthermore, the vast majority of anti-myeloma regimens contain a high load of dexamethasone, which may alter the metabolic profile substantially. Besides its anti-myeloma effect, dexamethasone may induce disturbances in lipid and glucose metabolism, as well [152]. Therefore, the metabolic phenotype of patients with relapsed/refractory MM may vary significantly and reflect the metabolic imprint of previous lines of therapy.

## 14. Conclusions

In conclusion, MM is a heterogeneous disease with several therapeutic options that can prolong patients’ survival. Despite the incorporation of novel agents over the past few years, the underlying metabolic reprogramming through hypoxia and increased lactate enhances tumor growth and survival and supports drug resistance. Myeloma cells compete with immune cells with regards to their metabolic needs and this overlapping interaction needs to be further investigated. Metabolic disorders have an important impact in the care of patients with MM. Importantly, prognostic models incorporating metabolic indices may improve patient risk stratification in addition to the well-established revised ISS [23]. A further insight will explain tumor growth and progression, and probably identify predictive and prognostic biomarkers for the management of multiple myeloma. Glucose is the most important energy compound and is essential for all regular functions. Therefore, it is believed that targeting the aspects of glucose metabolism mentioned above could lead to novel therapeutic options. More specifically myeloma cells mainly enhance glycolysis and lactate production instead of TCA cycle activation. This results in tumor growth, myeloma survival and, finally, chemoresistance. When glucose metabolism is inhibited post exposure to therapeutic agents such as proteasome inhibitors, the myeloma cells become resistant, and their energy needs depend mainly on glutamine and thus are less vulnerable to apoptosis [153]. When glutamine is the main energy compound used, it also affects the bone marrow microenvironment.

The combination of antimyeloma treatment with agents targeting the relevant metabolic pathways could be a potential future strategy to cure the disease. Targeting the GLUT and MCT transporters, IGF-1, FAS, ETC and OXPHOS need to be further explored as potential anti-myeloma therapeutic strategies. Ideally, future preclinical and clinical studies will help to elucidate metabolism’s role in myeloma development and progression and may lead to the discovery of novel therapies for patients suffering from this disorder. Metabolic syndrome is more common in patients with MM and the relevant disorders seem to affect the clinical outcomes of the disease. If this phenomenon is an epidemiological association or has pathophysiological links—possibly bidirectional—or it is the effect of medications used for the MM remains to be fully elucidated. Until then, increased awareness of the presence of metabolic syndrome or its components in patients with MM is advisable. BMI should be estimated, waist circumference should be measured, and glycemic status, lipid profile and blood pressure should be periodically monitored.

## Data Availability

Not applicable.
